# Pediatricians’ Perspectives on Task Shifting in Pediatric Care: A Nationwide Survey in Japan

**DOI:** 10.3390/healthcare13141764

**Published:** 2025-07-21

**Authors:** Masatoshi Ishikawa, Ryoma Seto, Michiko Oguro, Yoshino Sato

**Affiliations:** Research Institute, Tokyo Healthcare University, Higashi Gotanda 4-1-17, Shinagawa, Tokyo 141-8648, Japan

**Keywords:** pediatricians, physician workload, task shifting, work style reform, Japan

## Abstract

**Background/Objectives**: In Japan, task shifting reduces the working hours of pediatricians, who face excessive workloads. The status of task shifting under the Ministry of Health, Labor, and Welfare’s reforms remains unclear. This study aimed to evaluate the current status and barriers of task shifting in pediatric care in Japan. **Methods**: A questionnaire survey was conducted among pediatricians working in hospitals in Japan. The results were compared with those from 2020. **Results**: Questionnaires were sent to 835 hospitals, and valid responses were received from 815 pediatricians in 316 hospitals (response rate: 37.8%). The largest group (31.0%) was 40–49 years, and 34.4% of the participants were women. Among the items surveyed, most pediatricians indicated “shifted” in “Patient transfer (transporting between hospitals using an ambulance)” and “Intravenous injection of antibiotics.” Most physicians believed task shifting improved care quality; 10.3% felt it worsened. The most common estimate for daily working hour reduction due to task shifting was “1 to <2 h” (44.9%). Precisely 15.8% of pediatricians believed that task shifting had “not progressed at all,” with rural areas and non-university hospitals showing lower task-shifting implementation. National university hospitals had a higher likelihood of task shifting than public hospitals. No significant associations were observed for the total hospital bed count or the number of full-time pediatricians. **Conclusions**: Task shifting in pediatric care remains underdeveloped. While many pediatricians support the concept and report modest reductions in working hours, actual implementation remains limited. Future efforts must address systemic, institutional, and regulatory challenges to facilitate meaningful task redistribution and improve healthcare delivery.

## 1. Introduction

According to the World Health Organization (WHO), task shifting “presents a viable solution for improving health care coverage by making more efficient use of the human resources already available and by quickly increasing capacity while training and retention programs are expanded” [1].

The effectiveness of task shifting in improving healthcare efficiency has been demonstrated in various countries [2]. Its benefits have been proven in different aspects of medical care, such as non-communicable diseases [3], human immunodeficiency virus/acquired immune deficiency syndrome [4], primary care [5], and pediatric care [6]. In the field of maternal and newborn healthcare, the WHO issued recommendations on task shifting in 2012 [7,8]. Task shifting has become a beneficial strategy for making effective use of limited healthcare resources.

Task shifting is practiced in diverse clinical contexts globally. For example, nurse practitioners in the UK manage chronic disease and routine consultations; in sub-Saharan Africa, nurses deliver antiretroviral therapy; and in Nordic countries, midwives perform routine prenatal screenings [2,4,5]. These cases highlight the feasibility of safely delegating care tasks across professions.

Working hours in Japan are among the highest in Organisation for Economic Co-operation and Development countries [8]. In the healthcare sector, particularly among pediatricians, excessive working hours have been linked to medical errors, burnout, and reduced quality of patient care [9]. These conditions underscore the urgent need for systemic reforms such as task shifting.

In 2016, Japan’s Ministry of Health, Labor, and Welfare (MHLW) established a “Study Group on the Work Style Reform for Doctors” to consider measures for promoting work style reforms for doctors. In 2019, the Ministry announced “Urgent Measures for Reducing Doctors’ Working Hours” [10]. The proposal included promoting task shifting as a key strategy for reducing doctors’ working hours.

The WHO issued formal guidelines in 2008 and 2012 regarding task shifting, emphasizing that such delegation must be accompanied by appropriate training, supervision, and regulatory safeguards [1,7]. In Japan, the MHLW also issued recommendations in its 2019 report, outlining allowable procedures and responsible personnel for task reallocation [10].

Specifically, it involves transferring tasks, such as preliminary questioning during initial consultation, briefing on examination procedures and hospitalization, briefing on medications and providing guidance on their use, performing venous blood sampling, administering intravenous injections, securing intravenous lines, placing urinary catheters, making proxy entries into medical certificates, and patient transfers to other health care professionals [10].

To promote task shifting from pediatricians to other professionals, it is necessary to identify specific tasks for shifting, taking into account the characteristics of the medical specialty, and examine the impact of promoting task shifting on the quality of healthcare and working hours. Therefore, in 2020, we conducted a survey to investigate the intentions of pediatricians, which clarified the aforementioned aspects [11].

Regarding the status of task shifting for individual tasks, more than half of the pediatricians indicated only “Medication guidance” and “Intravenous injection of antibiotics, etc.” as tasks that had “shifted,” indicating that task shifting had not progressed in many areas [11]. Subsequently, the MHLW has facilitated the reform of physicians’ working conditions, and task shifting is believed to be advancing in pediatric healthcare as well. However, the progress of this shift is not yet clear.

The aims of this study were to identify the tasks that should be shifted in pediatric healthcare, collate opinions on the impact of task shifting on the quality of care and working hours, and compare the results with those from the 2020 survey to examine the progress of task shifting and suggest healthcare policies to promote task shifting.

## 2. Materials and Methods

### 2.1. Study Participants

This cross-sectional study was conducted to obtain information from pediatricians regarding their opinions on task shifting. This study targeted 835 hospitals in Japan with pediatric departments. The survey respondents were pediatricians, including both attending physicians and pediatric residents, who were actively practicing in clinical pediatric care [12]. This study targeted 835 hospitals registered in the Hospital Bed Function Reporting System, which is publicly maintained by the MHLW. These hospitals were selected to represent all secondary medical care areas nationwide, ensuring geographic and institutional diversity. In June 2024, survey requests were sent to the persons in charge of the pediatrics departments of these hospitals, and a web-based questionnaire survey was conducted. The full content of the questionnaire used in this study is provided as Supplementary Material (Supplementary File S1) to enhance transparency and reproducibility.

The respondents’ characteristics (sex, age, qualifications, job title, working hours per week, foundational entity of the hospital, total number of beds, number of pediatricians, and regional classification) were obtained (Table 1).

Age was categorized into five groups: under 30, 30s, 40s, 50s, and ≥60 years. Job title was categorized into four groups: department head, staff, resident, and others. The working hours per week were categorized into five groups: <40 h, 40–60 h, 60–80 h, 80–100 h, and ≥100 h. The foundational entity of the hospital was categorized into four groups: public and governmental, national universities, private universities, and private. The total number of beds was categorized into five groups: <200, 200–400, 400–600, 600–800, and >800 beds. Regional characteristics were classified, based on the population size and density in the 344 secondary medical care areas, into three categories: urban, intermediate, and rural areas [13].

### 2.2. Statistical Analysis

**Figure 1:**
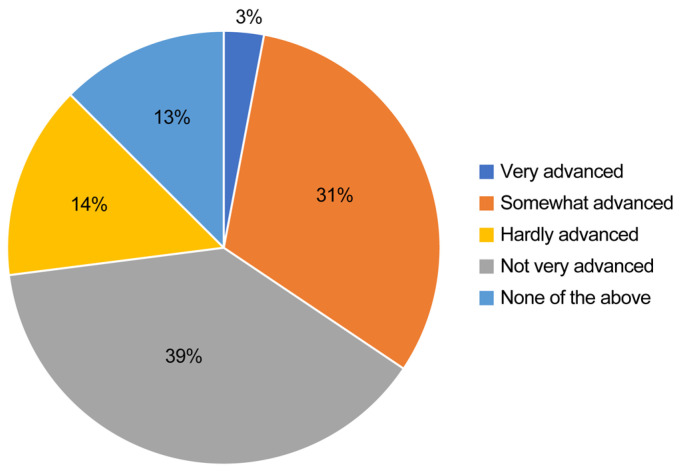
Chart showing the progress in task shifting in hospitals where the nurses work.

The responses to the question regarding whether task shifting is advanced at the respondent’s workplace (very advanced, somewhat advanced, not very advanced, not progressed at all, and none of the above) were visualized in Figure 1. Additionally, multivariate logistic regression analysis was performed, with the dependent variable being the physicians who answered “Not progressed at all” and the explanatory variables being physician attributes (foundational entity of hospital, total number of beds, number of pediatricians, and regional classification) to analyze the characteristics of physicians who believe that task shifting has not progressed at all at their workplace.

Responses to the questions regarding the implementation status of task shifting for individual tasks, as well as the opinions on whether the task should be shifted if not yet implemented (shifted, should be partially shifted in the future, should be extensively shifted in the future, should not be shifted in the future, none of the above), are described.

For individual tasks, items that were not specific to pediatrics were based on the MHLW’s “Urgent Measures for Reducing Doctors’ Working Hours” [14]. For items specific to pediatrics, the opinions of the committee of the Japan Pediatric Society, to which the co-researcher belongs, were referenced [15].

**Figure 2:**
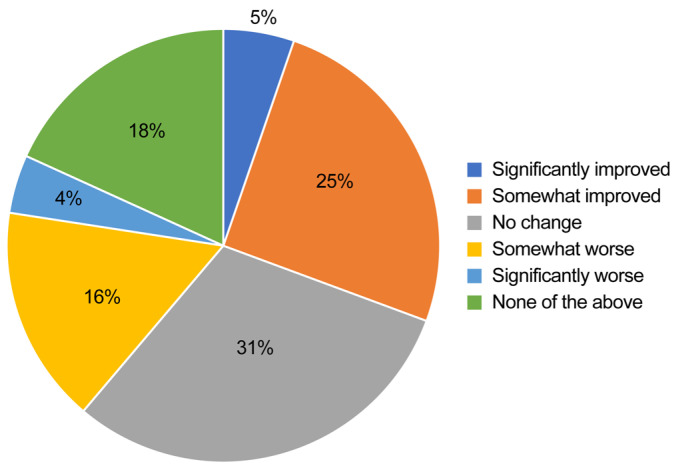
Chart showing the impact of task shifting on the quality of healthcare.

**Figure 3:**
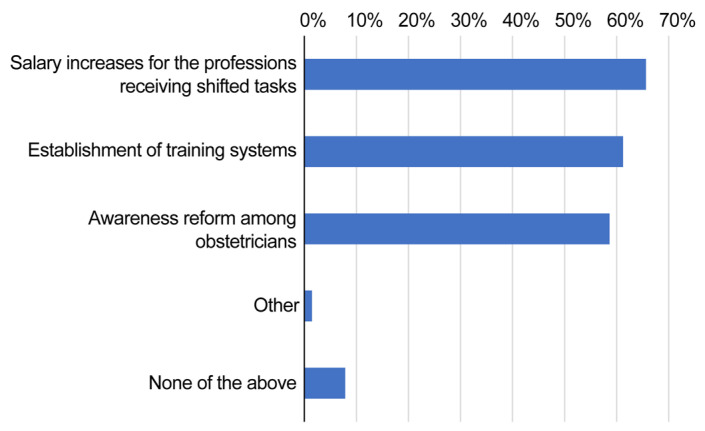
Chart showing what nursing personnel think is necessary to promote task shifting.

Figures were created to show the impact of task shifting on the quality of healthcare (significantly improved, somewhat improved, unknown, somewhat worse, significantly worse, none of the above) and the distribution of the amount of daily working hours believed to be reducible by task shifting (<1 h, 1–2 h, 2–3 h, 3–4 h, ≥4 h, unknown) (Figure 2 and Figure 3).

For the statistical analyses, *p* < 0.05 was considered significant. STATA 17.0 was used for all the statistical analyses.

### 2.3. Ethical Considerations

This study was approved by the Tokyo Healthcare University Research Ethics Committee for Human Studies (Approval Number: Kyo-023-24B), approval 26 February 2024. Written informed consent was obtained from all the participants. The study objective and measures to ensure secure data management were stated on the first page of the questionnaire. We also explained to potential participants that their involvement in the study was purely voluntary. The results were analyzed separately from the personal information to ensure anonymity and confidentiality.

## 3. Results

The questionnaire was sent to 835 hospitals, and valid responses were obtained from 815 pediatricians in 316 hospitals (response rate: 37.8%). The 815 pediatricians who responded represent 7.4% of the 11,030 pediatricians working in hospitals, as reported in the 2022 MHLW’s statistics on physicians, dentists, and pharmacists [16].

The respondents’ characteristics are shown in Table 1. There were 34.4% females, with the largest group, at 31.0%, being between 40 and 49 years old. Of the respondents, 80.2% were married. Among the sub-specialties, neurology was the most common, accounting for 12.8%, and the most common income level was greater than 14 million yen. Regarding weekly working hours, 31.7% worked between 50 and 60 h per week, which was the most common level. Regarding the foundational entity of the hospital, 63.1% worked in public hospitals, and 53.3% were employed in regional cities. The highest proportion of hospitals had between 400 and 600 beds.

When asked whether task shifting was advanced at their workplace, as shown in Figure 1, 2.5% of respondents answered “Very advanced,” and 33.4% answered “Somewhat advanced.” In contrast, 45.5% answered “Not very advanced,” and 15.8% answered “Not progressed at all.”

An analysis of the characteristics of the hospitals where the 15.8% of physicians who considered task shifting to be “Not progressed at all” revealed that compared to urban cities, hospitals in rural areas had a significantly lower likelihood of task shifting (0.53, 95% confidence interval [CI]: 0.35–0.80, *p* < 0.01). Although not statistically significant, national university hospitals had a higher likelihood (2.20, 95% CI: 0.96–5.06, *p* = 0.06) of task shifting than public hospitals. No significant associations were observed between the total number of beds and the number of full-time pediatricians, as shown in Table 2.

The implementation status of task shifting for individual tasks and opinions on whether to implement them if not already shifted are shown in Table 3.

For most respondents, the only tasks for which more than half answered “shifted” were “Patient transfer (transporting between hospitals using an ambulance)” and “Intravenous injection of antibiotics, etc.”

Regarding proxy entry, in all the items, the percentage of respondents who answered “shifted,” “should be extensively shifted in the future,” or “should be partially shifted in the future” exceeded 70%. For “Preparation of medical certificates and referral letters” and “Electronic medical chart entries,” the percentage of “shifted” responses exceeded 20%, indicating that a certain degree of task shifting had been implemented.

Regarding patient briefing, etc., the combined percentage of respondents who answered “shifted,” “should be extensively shifted in the future,” or “should be partially shifted in the future” exceeded 70% for all the items. For “Responding to telephone inquiries from patients and family” and “Patient transfer (transporting between hospitals using an ambulance),” the percentage of “shifted” responses exceeded 20%, indicating that a certain degree of task shifting had been implemented.

Regarding pediatric procedures, excluding “Vaccine administration (BCG)” and “Adjustment of ventilator settings (neonatal intensive care unit),” the combined percentage of responses for “shifted,” “should be extensively shifted in the future,” or “should be partially shifted in the future” exceeded 70%. Additionally, for “Venous blood sampling (other than newborns/infants),” “Intravenous injection of antibiotics, etc.,” “Specimen collection (stool, pharynx, nasopharynx, sputum, urine pack),” and “Specimen collection (newborn mass screening),” the percentage of “shifted” responses exceeded 20%, indicating that a certain degree of task shifting had been implemented. Regarding the impact of promoting task shifting on the quality of healthcare, as shown in Figure 2, 54.5% of doctors answered that the quality would improve somewhat or significantly, exceeding half of the respondents. Additionally, as shown in Figure 3, the most common response regarding the potential reduction in daily working hours due to task shifting was “1–2 h,” with 44.9% of the respondents selecting this option.

## 4. Discussion

The study findings suggest that although most pediatricians acknowledge the potential of task shifting to reduce workloads and enhance care quality, its implementation remains uneven across institutions, likely due to entrenched professional hierarchies and institutional resistance [17,18]. This observation is consistent with institutional theory, which explains how established professional hierarchies in medical systems resist change, particularly when reforms challenge traditional power dynamics. In the Japanese context, such resistance is likely amplified by the strong dominance of physician-centered governance within hospitals, where this model of care is deeply embedded.

We conducted a survey on task shifting among pediatricians working at hospitals nationwide. The results showed that 35.8% of the respondents believed that task shifting was “Very advanced” or “Somewhat advanced,” while 15.8% thought it had “Not progressed at all.” The gap in opinions among doctors may be attributed to differences in the progress of work style reforms at the hospitals where they are employed, as well as varying opinions among pediatricians on how task shifting should be implemented in pediatric healthcare. Overall, there was a tendency for many to believe that task shifting has not significantly progressed.

Among the doctors who answered “Not progressed at all” regarding task shifting, there was a significant tendency for a higher odds ratio in national university hospitals (compared to public hospitals). Regarding regional classification, although not statistically significant, a higher odds ratio was observed in urban areas. Additionally, no significant associations were found with the total number of hospital beds or the number of full-time pediatricians.

In Japan, university hospital doctors are often overworked due to their involvement in education, clinical practice, and research. At the same time, some doctors are working without pay, highlighting poor working conditions [19,20]. In this study, we found that task shifting might not be progressing in national and public universities. The situation where young, unpaid doctors provide cheap labor [21] could be a reason for the lack of progress in task shifting. This trend aligns with the findings from the 2020 survey, suggesting that the “physician work style reform” may not be progressing [11].

Regarding regional differences, there are reportedly more doctors in large urban areas [22], and as such, in urban areas with a high number of doctors, task shifting may not progress as much. In contrast, in small cities or rural areas, task shifting may progress more, potentially contributing to alleviating the shortage of doctors.

Regarding task shifting for individual tasks, as shown in Table 3, most pediatricians responded that only two items, “Patient transfer (transporting between hospitals using an ambulance)” and “Intravenous injection of antibiotics, etc.,” “shifted.” These findings were similar to the 2020 survey [11], indicating that task shifting has not progressed significantly.

Despite policy efforts since 2019, our results suggest that task shifting has not been widely implemented in practice. Structural barriers, such as the absence of nationally standardized delegation protocols, a lack of interprofessional education frameworks in medical training, and institutional inertia, continue to hinder task shifting in practice [1].

In contrast to countries such as the UK, where nurse practitioners are legally authorized to prescribe medications and manage chronic diseases, or Canada, where interdisciplinary pediatric teams are widespread, Japan’s approach to task shifting remains cautious and fragmented [23,24]. In comparison, countries such as the UK and Canada have institutionalized task sharing through expanded nursing roles supported by legislation and standardized certification. Japan’s physician-centered culture and hospital governance may require transformation to facilitate similar progress [23]. To promote broader adoption of task shifting, policymakers should consider introducing targeted legislative changes, interprofessional training programs, and financial incentives.

Even for tasks like “Initial consultation interviews (preliminary questioning)” or “Briefing test procedures,” which were classified as “should not be shifted” in the materials from the Japan Pediatric Society [15], over 70% of the pediatricians who responded considered that they “should be shifted in the future.” While individual circumstances must be taken into account, this suggests that there may be more room for advancing task shifting beyond what society currently envisions.

Regarding the impact of task shifting on the quality of healthcare as perceived by pediatricians, 54.5% of physicians responded that the quality of care would improve, while only 10.2% believed it would worsen. Although it is important to avoid any decline in the quality of care due to task shifting, at least most respondents in this survey did not hold that view.

While nearly 45% of pediatricians anticipated a 1–2 h daily reduction in workload due to task shifting, this expectation may reflect aspirational thinking rather than observed outcomes. According to implementation research, such optimism often precedes the necessary organizational restructuring required for actual impact. This may reflect optimism about task shifting’s potential rather than evidence of current structural changes. Future studies should investigate whether these perceived gains translate into measurable reductions in labor hours. If task shifting leads to a reduction of 1–2 h in daily working hours, it is expected that labor hours could improve to a level closer to the legally mandated 40-h workweek.

By combining nationwide data with comparative analysis data from 2020, this study empirically demonstrated that although task shifting remains limited in scope, it holds measurable potential to alleviate excessive working hours and modestly improve care delivery, especially if policy frameworks are clarified and operational support is provided. A key strength of this study lies in its nationwide scope and the large number of participating institutions, which offer a comprehensive view of current practices and physician attitudes toward task shifting across Japan. By comparing these results with those of a prior national survey conducted in 2020, this study also captures trends over time, allowing for the assessment of policy implementation effectiveness and institutional change.

From an educational perspective, the findings highlight the urgent need to integrate interprofessional collaboration and task delegation principles into the training curricula for both pediatricians and allied health professionals, particularly nurses and medical assistants. This may involve formal modules on clinical task distribution, communication across disciplines, and shared accountability in patient care.

In practical terms, the study underscores the importance of systemic interventions, such as the development of standardized training and certification frameworks for non-physician staff, hospital-level workflow redesign, and regulatory reforms that clearly delineate delegable tasks. These actions can facilitate safe and efficient redistribution of responsibilities, helping ensure sustainability in pediatric healthcare delivery amid workforce constraints.

This study has several limitations. First, although we obtained responses from 815 pediatricians across 316 hospitals, this represents only 7.4% of hospital-based pediatricians nationwide, which may limit the generalizability of our findings. Second, participation in the survey was voluntary, introducing the possibility of selection bias. The respondents may have been more interested in physician workstyle reform or task shifting, potentially skewing the results toward more favorable or critical perspectives. Third, we employed a non-random sampling strategy, as the hospitals were selected from the MHLW’s publicly available database, and the responses were dependent on departmental cooperation. Therefore, the sample may not fully reflect the entire institutional diversity of pediatric care settings across Japan. Fourth, the use of self-reported data may have introduced recall bias or social desirability bias, particularly regarding the perceived effects of task shifting on quality of care or workload reduction. Fifth, the cross-sectional design of the study precludes any inference of causality between institutional characteristics and the progress of task shifting. Longitudinal studies are needed to determine temporal trends and causal relationships. Lastly, although the data were obtained from individual physicians, the hospital was not the unit of analysis, which may have led to an underestimation or overestimation of task-shifting implementation at the institutional level. Future studies should consider multi-level modeling to account for institutional clustering and variation.

## 5. Conclusions

This nationwide survey indicates persistent stagnation in the progress of task shifting in pediatric care, despite official policy endorsements. The gap between pediatricians’ favorable attitudes and actual implementation highlights systemic inertia and the need for coordinated action. Physician work reform is a global issue [7]. To promote task shifting in Japanese pediatric healthcare, it is essential to continue advancing policy guidance in terms of systems and education while considering the opinions of pediatricians and the peculiarities of other healthcare professions.

Drawing from international best practices, we propose the following multi-level policy actions: (1) The Ministry of Health should establish clear and legally enforceable task delegation frameworks as seen in the UK; (2) medical associations should lead the development of accredited training and certification programs, similar to the Canadian model; and (3) hospitals should receive financial and organizational incentives to implement team-based care models that support safe and efficient task shifting.

## Figures and Tables

**Table 1:** Respondents’ characteristics.

No. of Respondents	2043	
Sex		
Female	2041	99.9%
Male	2	0.1%
Age, years		
<30	434	21.2%
30s	503	24.6%
40s	602	29.5%
50s	443	21.7%
≥60s	61	3.0%
Qualifications		
Midwife	1640	80.3%
Registered nurse	1463	71.6%
Licensed practical nurse	99	4.8%
Advanced practice midwife	397	19.4%
Job title		
Staff	1462	71.6%
Chief	360	17.6%
Head nurse	180	8.8%
Others	41	2.0%
Foundational entity of employer		
Public	1262	61.8%
Private	551	27.0%
Public university	131	6.4%
Private university	99	4.8%
Employer’s total no. of beds		
<200 beds	176	8.6%
≥200 to <400 beds	632	30.9%
≥400 to <600 beds	753	36.9%
≥600 to <800 beds	294	14.4%
≥800 beds	188	9.2%
Employer’s regional classification		
Urban	693	33.9%
Intermediate	1062	52.0%
Rural	288	14.1%
Number of full-time obstetricians		
<5	841	41.2%
5–9	858	42.0%
≥10	293	14.3%
Unknown	51	2.5%
Number of full-time midwives		
<10	389	19.0%
10–20	763	37.3%
20–30	450	22.0%
≥30	384	18.8%
Unknown	57	2.8%
Number of full-time advanced practice midwives		
<5	803	39.3%
5–9	564	27.6%
≥10	222	10.9%
Unknown	454	22.2%
Annual delivery count		
None	45	2.2%
<200	509	24.9%
200–400	714	34.9%
≥400	642	31.4%
Unknown	133	6.5%
Number of inpatient midwifery cases per year		
None	1468	71.9%
<200	302	14.8%
200–400	43	2.1%
≥400	13	0.6%
Unknown	217	10.6%
Number of outpatient midwifery cases per year		
None	572	28.0%
<200	733	35.9%
200–400	253	12.4%
≥400	274	13.4%
Unknown	211	10.3%

**Table 2:** Association between task shifting and respondents’ characteristics.

	OR	95% CI	*p*-Value
Foundational entity of employer			
Public	Reference		
National university	1.15	0.56–2.37	0.70
Private university	0.43	0.05–3.99	0.46
Private	2.13	0.35–12.90	0.41
Total no. of beds			
<200 beds	Reference		
≥200 to <400 beds	0.55	0.23–1.33	0.19
≥400 to <600 beds	0.35	0.14–0.84	0.02
≥600 to <800 beds	0.19	0.05–0.73	0.02
≥800 beds	0.34	0.06–1.87	0.22
Workplace			
Urban	Reference		
Intermediate	1.04	0.53–2.04	0.90
Rural	1.69	0.64–4.49	0.29
Number of full-time obstetricians			
<5	Reference		
5–9	1.19	0.55–2.58	0.65
≥10	2.25	0.77–6.60	0.14
Unknown	0.38	0.04–3.96	0.42
Number of full-time midwives			
<10	Reference		
10–20	0.38	0.15–0.96	0.04
20–30	0.73	0.25–2.11	0.57
≥30	0.41	0.12–1.42	0.16
Unknown	1.46	0.29–7.27	0.64
Number of full-time advanced practice midwives			
<5	Reference		
5–9	0.69	0.32–1.49	0.35
≥10	0.53	0.19–1.49	0.23
Unknown	0.65	0.28–1.48	0.30
Annual delivery count			
None	Reference		
<200	0.33	0.07–1.65	0.18
200–400	0.37	0.07–2.06	0.26
≥400	0.71	0.12–4.15	0.71
Unknown	0.28	0.04–2.20	0.23
Number of inpatient midwifery cases per year			
None	Reference		
<200	4.02	2.02–7.99	<0.01
200–400	7.60	2.22–26.09	<0.01
≥400	2.53	0.47–13.54	0.28
Unknown	2.57	1.01–6.54	0.05
Number of outpatient midwifery cases per year			
None	Reference		
<200	2.13	0.79–5.71	0.13
200–400	1.90	0.55–6.63	0.31
≥400	6.70	2.36–19.01	<0.01
Unknown	2.88	0.81–10.22	0.10

OR, odds ratio; CI, confidence interval.

**Table 3:** Opinions regarding the implementation of task shifting.

1. Proxy entry tasks								
Task	Shifted	Should be shifted in the future	Should not be shifted	Neither	Shifted	Should be shifted in the future	Should not be shifted	Neither
Initial consultation interviews (preliminary questioning)	960	221	425	437	47.0%	10.8%	20.8%	21.4%
Order entry for tests, prescriptions, and procedures	296	301	1011	435	14.5%	14.7%	49.5%	21.3%
Hospitalization and surgery bookings	272	370	984	417	13.3%	18.1%	48.2%	20.4%
Preparation of medical certificates and referral letters	263	267	1175	338	12.9%	13.1%	57.5%	16.5%
Preparation of discharge summaries	299	335	949	460	14.6%	16.4%	46.5%	22.5%
Electronic medical chart entries	261	261	1035	486	12.8%	12.8%	50.7%	23.8%
Case registrations (e.g., cancer registration)	141	372	963	567	6.9%	18.2%	47.1%	27.8%
2. Patient briefings and general procedures								
Task	Shifted	Should be shifted in the future	Should not be shifted	Neither	Shifted	Should be shifted in the future	Should not be shifted	Neither
Responding to telephone inquiries from patients	1048	257	257	481	51.3%	12.6%	12.6%	23.5%
Briefings using leaflets and video clips	973	440	178	452	47.6%	21.5%	8.7%	22.1%
Utilizing online medical consultations	75	358	613	997	3.7%	17.5%	30.0%	48.8%
Patient transfer (from operating room to hospital room, etc.)	1297	245	178	323	63.5%	12.0%	8.7%	15.8%
Sampling blood culture specimens	1277	242	223	301	62.5%	11.8%	10.9%	14.7%
Securing contrast agent lines	1230	224	202	387	60.2%	11.0%	9.9%	18.9%
Securing chemotherapy lines	800	270	455	518	39.2%	13.2%	22.3%	25.4%
3. Obstetric-specific procedures								
Task	Shifted	Should be shifted in the future	Should not be shifted	Neither	Shifted	Should be shifted in the future	Should not be shifted	Neither
Routine fetal ultrasound	244	326	836	637	11.9%	16.0%	40.9%	31.2%
Screenings during prenatal check-ups	221	174	1287	361	10.8%	8.5%	63.0%	17.7%
Prescribing routine medications	209	593	841	400	10.2%	29.0%	41.2%	19.6%
Internal examinations during labor onset or rupture of membranes	1324	169	197	353	64.8%	8.3%	9.6%	17.3%
Starting labor-inducing drugs for weak contractions	372	180	1150	341	18.2%	8.8%	56.3%	16.7%
Adjustment of labor-inducing drugs	1024	181	527	311	50.1%	8.9%	25.8%	15.2%
Episiotomy and perineal suturing	67	217	1354	405	3.3%	10.6%	66.3%	19.8%
Bimanual uterine compression	120	212	1194	517	5.9%	10.4%	58.4%	25.3%
One-month postpartum check-up	143	242	1166	492	7.0%	11.8%	57.1%	24.1%
4. Surgical procedures in obstetrics and gynecology								
Task	Shifted	Should be shifted in the future	Should not be shifted	Neither	Shifted	Should be shifted in the future	Should not be shifted	Neither
Assistant in obstetric and gynecological surgeries	90	174	1318	461	4.4%	8.5%	64.5%	22.6%
Intraoperative anesthesia, respiratory, and circulatory management	439	107	1205	292	21.5%	5.2%	59.0%	14.3%
Postoperative drain management and removal	172	217	1203	451	8.4%	10.6%	58.9%	22.1%
Postoperative CV removal and PICC insertion	157	184	1300	402	7.7%	9.0%	63.6%	19.7%
Postoperative wound management (cleaning, suturing, and staple removal)	99	244	1262	438	4.8%	11.9%	61.8%	21.4%

PICC, peripherally inserted central catheter.

## Data Availability

Due to the nature of this research, the study participants did not consent to their data being shared publicly, so supporting data are not available.

## Supplementary Materials

The following supporting information can be downloaded at https://www.mdpi.com/article/10.3390/healthcare13141764/s1, Figure S1: Survey Questionnaire (English Translation).

## Abbreviations

The following abbreviations are used in this manuscript:

WHO	World Health Organization
