# Supplementary material for: Pediatricians’ Perspectives on Task Shifting in Pediatric Care: A Nationwide Survey in Japan
Source: Healthcare (Basel). 2025 Jul 21;13(14):1764. doi: 10.3390/healthcare13141764 (PMC12294679; doi:10.3390/healthcare13141764)
Supplement: Supplementary file 1 [file healthcare-13-01764-s001.zip › healthcare-3658839-supplementary.pdf]

☑ **Supplementary Material S1: Survey Questionnaire (English Translation).**

Title: Nationwide Survey on Task Shifting in Pediatric Care (2024)

Target: Pediatricians working in hospitals across Japan

Mode of Delivery: Web-based (Google Forms)

Estimated Completion Time: ~10 minutes

Section A: Respondent Characteristics

1. What is your gender?
   □ Male □ Female □ Prefer not to answer
2. What is your age group?
   □ Under 30 □ 30–39 □ 40–49 □ 50–59 □ 60 and above
3. What is your current title?
   □ Department Head □ Staff Physician □ Resident □ Other (please specify)
4. What is your average working time per week?
   □ Less than 40 h □ 40–60 h □ 60–80 h □ 80–100 h □ More than 100 h
5. What is the type of institution where you work?
   □ National University Hospital □ Public Hospital □ Private University Hospital □ Private Hospital
6. What is the number of pediatricians at your hospital?
   (Free text box)
7. What is the regional classification of your hospital?
   □ Urban □ Intermediate □ Rural

Section B: Perception of Task Shifting

8. To what extent is task shifting implemented at your institution?
   □ Very advanced □ Somewhat advanced □ Not very advanced □ Not progressed at all □ Not applicable
9. In your opinion, what is the impact of task shifting on the **quality of care**?
   □ Significantly improved □ Somewhat improved □ No change □ Somewhat worsened □ Significantly worsened □ Not sure
10. In your estimation, how much **daily working time** is reduced due to task shifting?
    □ Less than 1 hour □ 1–2 hours □ 2–3 hours □ 3–4 hours □ More than 4 hours □ Unknown

---

Section C: Task-Specific Implementation and Opinions

For each of the following tasks, please select the most applicable status.

Instructions: Select one for each row → ◻ Already shifted ◻ Should be partially shifted in future ◻ Should be extensively shifted ◻ Should not be shifted ◻ Not applicable

Task	Status
Preliminary questioning during initial consultation	
Providing test explanations using video/leaflets	
Responding to telephone inquiries	
Transferring patients between departments/hospitals	
Venous blood sampling (excluding neonates)	
Intravenous injection of antibiotics	
Entering orders for tests and prescriptions	
Writing medical certificates and referral letters	
Discharge summaries	
Case registration (e.g., cancer registry)	

Final Section: Free Comment

11. Please share any additional thoughts you have regarding task shifting in pediatric care: ____

📝 **Notes:**

- This questionnaire was originally developed in Japanese and translated into English for international review purposes.
- Items were developed with reference to MHLW guidelines and prior studies on physician workstyle reform.
- Full Japanese version is available upon request.
